# Challenges and opportunities for the implementation of virological testing in resource-limited settings

**DOI:** 10.7448/IAS.15.2.17324

**Published:** 2012-10-09

**Authors:** Teri Roberts, Helen Bygrave, Emmanuel Fajardo, Nathan Ford

**Affiliations:** 1 Médecins Sans Frontières Access Campaign, Geneva, Switzerland; 2 Médecins Sans Frontières Southern African Medical Unit, Cape Town, South Africa; 3 Centre for Infectious Disease Epidemiology and Research, University of Cape Town, South Africa

**Keywords:** viral load, virological, dried blood spots, resource-limited settings, anti-retroviral therapy, monitoring

## Abstract

Though the advantages of routine virological monitoring for patients on anti-retroviral therapy have been established, cost and complexity limit its full implementation. Monitoring is important for diagnosing virological failure early on, before the development of drug resistance mutations, and to trigger early adherence interventions. Simple and cost-effective viral load tests that facilitate simplification and decentralization of testing and strategies, such as the use of dried blood spots and pooled sample testing, which further aid simplification, are becoming available. In addition, replacing immunological monitoring with virological monitoring in non-viremic patients in a phased manner will reduce the costs associated with dual immuno-virological monitoring. Going forward, the simplification of testing paired with price reducing strategies that will allow for healthy competition between multiple manufacturers will enable the implementation of viral load testing in resource-poor settings. It is important that future HIV and AIDS treatment guidelines provide clear recommendations for routine virological monitoring and that governments and donors fund the implementation of accurate and operationally proven testing platforms in a comprehensive manner.

## Introduction

The benefits of virological monitoring for patients on anti-retroviral therapy (ART) are well established and include the ability to diagnose adherence problems and treatment failure, and optimize therapy to support reduced transmission [1–3]. However, there are a number of access barriers to viral load in resource-limited settings, including high cost, technical complexity and difficulties with sample transport from the periphery and quality control. The result is that, while viral load testing is the standard of care for patients in rich countries, routine virological monitoring is rarely available in most high-HIV prevalence settings. A recent survey across 23 low-resource countries revealed that national virological testing was available only for confirmation of treatment failure in Kenya and for routine treatment monitoring in Brazil, Botswana and South Africa [4].

Evidence of the benefit of viral load monitoring at the population level is mixed. Trials evaluating the short-term benefit of virological monitoring against clinical endpoints, which are considered delayed outcomes, have concluded no major benefit over-and-above clinico-immunological monitoring [5–7]. However, longer term observational studies have shown that clinico-immunological monitoring is inaccurate [3,8–10]. Furthermore, while it is well accepted that mortality follows a CD4 decline on treatment [11], both viremia copy-years and cross-sectional virological measurements can independently predict all-cause mortality as well [12]. Support for simpler, more affordable and more cost-effective technologies is growing [2–13], partly fuelled by a growing concern that unchecked viremia could lead to the development and transmission of drug resistance [14,15].

Recognizing these multiple benefits, the latest World Health Organization (WHO) guidelines for ART in resource-limited settings, issued in 2010, recommend that all countries begin to phase-in viral load monitoring. This viewpoint article provides an overview of current implementation barriers to viral load testing in resource-limited settings and provides some practical recommendations for increasing capacity for routine virological monitoring in low- and middle-income countries.

## Importance of routine virological monitoring

### Diagnose early virological failure on ART

Effective treatment should suppress viral replication. A measurable viral load is, therefore, a very accurate measure of unsuccessful treatment. The WHO defines virological failure as a viral load above 5000 copies/ml and recommends that virological monitoring be performed biannually [16]. Frequent monitoring enables the diagnosis of virological failure before the development of drug resistance mutations, which would ultimately lead to treatment failure and allow for possible viral transmission [17].

In the absence of virological monitoring, immunological monitoring by CD4 count change is recommended. WHO guidelines define immunological failure as a CD4 count falling to or below the baseline value, or a 50% fall from the on-treatment peak or persistent CD4 values below 100 cells/µl [16]. However, CD4 testing has a poor accuracy and low positive predictive value in both adults [10,18] and children [8] for diagnosing treatment failure. Thus, viral load remains the gold standard.

### Discriminate between poor adherence and treatment failure

Unsuccessful treatment, leading to virological failure, may be due to a number of reasons, including drug interactions, malabsorption and poor adherence [19–21]. While viral load has been seen as a tool to diagnose failure, the main benefit is the prevention of treatment failure by identifying patients in need of intensive adherence counselling. The WHO guidelines define treatment failure as *persistent* virological failure [16], the first episode of virological failure typically being followed by a period of intensive adherence counselling and support, followed by a second viral load. If virological failure persists, and there has not been a significant (>0.5 log) drop in viral load, treatment failure is diagnosed, with a consequential regimen switch. However, published data indicate that, in the majority of cases, viral suppression is achieved after intensive adherence counselling [20–23]. Early good adherence is predictive of long-term virological suppression [20], and there is some evidence that virological monitoring, if done soon after treatment initiation (i.e. at three months) leads to better outcomes by flagging those patients in need of adherence counselling [24].

Once non-adherence has been ruled out, persistent viremia indicates treatment failure and the need for an appropriate treatment switch. According to WHO guidelines, a persistent viral load above 5000 copies/ml confirms treatment failure [16], although some countries, such as South Africa and Zambia, have adopted a lower-level threshold of 1000 copies/ml [10,17]. Drug resistance occurs when patients are kept on failing regimens at virological levels above 1000 copies/ml, limiting future treatment options [25]. European guidelines recommend that an ARV drug resistance test be performed at a viral load above 500 to 1000 copies/ml [26]; however, data from a European multicentre cohort study showed that 15.14% of test results were obtained at viral loads <1000 copies/ml and that, while the probability of mutations occurring below 500 copies/ml was lower, their presence might indicate the emergence of drug resistance and allow for an earlier preventative intervention [27]. Guideline revisions to favour lower thresholds may therefore be necessary.

Importantly, the poor accuracy and positive predictive value of clinico-immunological monitoring compared to virological monitoring for predicting treatment failure means that, without viral load testing, patients are either diagnosed very late or misdiagnosed completely, with the result that patients can be kept on a failing regimen or switched unnecessarily. Furthermore, when clinico-immunological criteria are used to diagnose treatment failure, extensive drug resistance occurs, limiting the use of future treatment options [28,29]. Virological monitoring is therefore necessary for the confirmation of both clinical and immunological failure and should ideally be used for the timely diagnosis of treatment failure, before clinical or immunological deterioration [10,18,30].

### Support treatment monitoring and optimization

The superiority of virological monitoring over clinico-immunological monitoring for diagnosing virological failure has multiple benefits beyond the initiation of adherence interventions and the appropriate switching of treatment regimens. Reducing the risk of virological failure through targeted adherence counselling and support prevents the development of drug resistance mutations, leading to the preserved use of affordable, fixed-dose, first-line drugs [1,31]. The benefit of using the diagnosis of virological failure as a means to intervene and prevent disease progression early has been shown in studies which found that patients without access to annual virological monitoring have poorer outcomes [32]. Virological monitoring can serve as an independent predictor of AIDS-defining events and mortality, even at CD4 counts above 350 cells/µl [33–35]. In some Western settings, it is recommended that patients are initiated on ART at high viral loads (above 100,000 copies/ml), regardless of CD4 count [26].

### Simplification of ART delivery

To scale up treatment to the millions of people in need, ART delivery needs to be made as simple as possible, in line with the public health approach to HIV treatment and care promoted by the WHO. The management of treatment failure is one area where simplification is becoming increasingly urgent. Detection of treatment failure using standard immunological definitions is poorly implemented in resource-limited settings. Only 1.6% of patients receiving treatment as part of HIV programmes supported by Médecins Sans Frontières (MSF) in 19 countries have been switched to second-line therapy, suggesting very poor levels of detection [36]. Calculating CD4 changes over time from paper records is a challenge for clinicians, especially in overburdened clinics. In contrast, routine virological monitoring provides a useful cross-sectional measurement of treatment efficacy, reducing the necessity to review historic data and facilitating appropriate clinical interventions (such as adherence counselling or regimen switching).

Having a test that clearly confirms virological suppression may also allow for less frequent clinical follow-up and further task shifting. Simplification of treatment monitoring using an annual clinical visit with review of the viral load could significantly reduce the number of clinical contacts required, having both a cost-saving effect and reducing the burden on patients and healthcare workers alike.

## Options for increasing access to virological testing

### Types of tests

An overview of current and pipeline tests for viral load has been provided elsewhere [37].

#### Molecular versus non-molecular testing

Viral load assays have traditionally been based on the amplification of nucleic acid using molecular techniques, such as real-time polymerase chain reaction (PCR). However, contamination with foreign nucleic acid or amplicon (PCR products) can cause false positive results, and great care must be taken to avoid cross-contamination of samples [38,39]. Moreover, precision pipetting is required to achieve an accurate result. One way to limit contamination and pipetting errors is to automate the process as far as possible, which is feasible with currently available technologies for sample preparation and subsequent amplification and detection. PCR products should be contained and disposed of or safely stored directly after the amplification and detection stage [40].

As an alternative, a non-molecular test, such as the ExaVir Load Assay (Cavidi, Uppsala, Sweden), may be used. This method relies on the detection of reverse transcriptase as a surrogate marker for HIV RNA using an ELISA-type technique routinely used even at district laboratory level [38]. A major advantage is that the enzyme is conserved across HIV-1 strains and is therefore subtype independent [41]. While inexpensive and easy to perform, this assay has a number of disadvantages, including the use of plasma as a sample type, lower through-put (the test takes two days to perform and a technician can only process a maximum of 180 samples/week compared to four hours processing and 800 samples/week for molecular tests); no automation, resulting in demand for hands-on time; and the inability of the manufacturer to supply controls (known HIV-positive and HIV-negative plasma must be supplied on-site for this purpose) [38,42].

Viral load may also be measured by quantifying the concentration of p24 antigen. This non-molecular test is cheaper and simpler than a test for HIV RNA [41]. While the WHO recommends the use of ultrasensitive p24 testing for early infant diagnosis, it is not considered sufficiently sensitive to serve as a treatment monitoring tool [38,43]. A further disadvantage is that there is only one test available for the ultrasensitive measurement of p24, manufactured by PerkinElmer (Waltham, USA), although it is not commercialized and may only be used for research purposes [37]. Given that the concentration of p24 has been found to correlate with HIV RNA and predicts clinical stages and mortality [44], further research into the use of p24 for treatment-monitoring purposes may be warranted in areas where HIV RNA testing is not available due to resource constraints.

#### Laboratory-based tests versus point-of-care devices

MSF has set up a molecular laboratory at the district level in Thyolo, Malawi, to offer viral load testing using the NucliSENS EasyQ HIV-1 v2.0 assay (bioMérieux, Marcy-l’Étoile, France). The NASBA-based technique was chosen due to the fact that the test has been validated on dried blood spots (DBS) [45,46], which was the chosen sample type. A number of logistical challenges were encountered during the setting up of this laboratory, including unsuitable laboratory infrastructure; unreliable power supply; unreliable water supply and provision of RNAse-free water; unreliable air-conditioning; non-adherence to cold chain transportation, especially at customs; inability to find local laboratory technicians with molecular biology expertise; and lack of in-country troubleshooting and maintenance services.

These findings are not unique to MSF and the two main implementation barriers to be overcome for facilitating access to viral load testing in resource-limited settings are cost and complexity [2]. The development of simpler laboratory-based tests, or even point-of-care devices, could therefore go a long way in solving these access problems, if prices are low enough. Current tests are not considered suitable for district laboratory settings because they are expensive and technically complex, requiring a large laboratory area and highly trained staff. Two exceptions may be the ExaVir Load Assay, a non-molecular, ELISA-based technique, and the Generic HIV-1 Viral Load Assay (Biocentric, Bandol, France), which has been developed by the Agence Nationale de Recherches sur le SIDA et les hepatites virales (ANRS) for resource-limited settings and has a small laboratory footprint [41–47]. Both tests may be performed at district laboratory level and are less costly than their counterparts, but still rely on medium to highly trained technicians [38–41].

There is a pipeline of devices that, if shown to be technically validated, cost-effective and field appropriate, will greatly enhance our ability to implement viral load testing in a decentralized approach, at point of service. The first products are predicted to be available from 2013. These include greatly simplified laboratory-based tests that can be used at district level, or in mini-laboratories set up alongside public health clinics, and automated, all-in-one, point-of-care tests that can be used at the clinic level by clinicians or even lay workers. A review of the pipeline has recently been published and will not be considered further here [48].

A number of operational challenges will have to be overcome during the implementation of these new devices, including the cost-effectiveness compared to centralized laboratory-based testing; the ability to meet through-put requirements; the effect on health service outcomes, such as staff work-load at the clinics, and number of transcription errors; and the effect on patient outcomes (morbidity, mortality and retention in care). In addition, staff should be adequately trained to acquire the appropriate sample and operate the instruments correctly, and strict quality control should be mandatory, even at decentralized facilities [39–49].

Options for roll-out of routine viral load may be considered in three tiers: (1) a centralized high through-put approach utilizing traditional platforms paired with DBS as a sample transport method; (2) simple, automated devices at district laboratory level; and (3) true point-of-care devices at individual clinic level. The choice will depend on the individual programme setting, cohort size, and whether the epidemic is generalized or concentrated. Where a decentralized approach to ART provision is implemented, careful consideration needs to be given to through-put requirements, feasibility of ensuring quality control and the cost-effectiveness of a true point-of-care test. These should be balanced against the need for an effective specimen collection and result delivery system in a centralized approach.

Experience of moving CD4 testing for ART initiation from a centralized laboratory to a point-of-care approach provides some insights about potential challenges for implementation of point-of-care viral load testing. Task shifting for performance of the test has proven feasible, with improved patient retention prior to ART initiation [50,51]. However, sampling errors have illustrated the importance of adequate staff training in the implementation of point-of-care CD4 testing [51–53].

## Approaches to a phased implementation

The benefits of providing routine viral load monitoring in resource-limited settings were recognized a decade ago [54], but, with the exception of South Africa and Botswana, widespread access to routine viral load testing in Africa is still a long way off. Nevertheless, a number of approaches have been recently piloted to support the WHO recommendation to phase in viral load testing. These are discussed in the subsequent sections.

### 

#### Dried blood spots

The sensitivity of molecular viral load testing is dependent on the volume of sample, and 1ml of plasma is usually recommended to achieve a sensitivity down to 50 viral copies/ml. The disadvantages of using plasma as a sample type are that whole venous blood must be drawn by a health professional and plasma must be separated from the whole blood within six hours of blood draw [40]. This is both impractical and unreliable in remote settings that are far from laboratories or where the clinics do not have a daily transport network for samples. Transportation of samples, in particular, remains one of the biggest challenges to viral load testing in resource-limited settings. One solution is to use DBS [55], where whole blood is pipetted onto filter paper, which is then stored, with desiccant, in an air-tight bag [42]. Whole blood may be taken from a finger or heel prick, for example, by trained lay workers. This overcomes the need for clinical staff or phlebotomists, and desiccated filter papers may be transported easily over long distances, without the need for a cold-chain or speedy delivery, with elution of the nucleic acid from the filter paper being the only extra laboratory step [40,42]. The preparation of DBS is commonly used in resource-limited settings as a sample type for early infant diagnosis and is therefore a familiar and well-established technique [42,56]. Genotypic resistance testing may also be performed from DBS [46].

There are two potential disadvantages to DBS. First, the small sample volume (50 to 100µl) results in poor sensitivity at lower viral loads below 3000 viral copies/ml [42,45], making it difficult to use a threshold of 1000 copies/ml to reliably diagnose virological failure [57]. Second, the use of whole blood rather than plasma means that pro-viral DNA and cellular RNA are amplified along with plasma viral RNA, artificially raising the viral load at lower values below 5000 copies/ml [58]. The latter may lead to a false diagnosis of virological failure with adverse clinical implications. The only technique currently available that is RNA specific is the NASBA technique, used in the NucliSENS Assay [42]. Alternatively, a DNAse pre-step, or DNase-containing filter paper, may be used to select for RNA [59]. Thus, the limits of DBS-based virological testing may be overcome by (1) raising the threshold for virological failure to 3000 copies/ml and (2) using RNA-specific techniques that select for viral RNA so that pro-viral DNA contamination may be avoided.

#### Pooled viral load testing

Pooled sample testing is a strategy to reduce the number of samples run by combining five to ten samples together [60–62]. If the pooled sample tests positive, an algorithm is then used to identify those individuals with a detectable viral load, or, failing this, the individual samples in the pool are tested individually. When less than a third of patients are viremic, negative predictive values are 100% at viral loads above 500 copies/ml.

Pooled viral load testing can reduce the number of individual tests required by up to 60%, without compromising on accuracy. Cost savings are significant, with one study from Mexico quoting a saving of up to $14,308 by a 30% reduction in individual testing [60], a study in San Diego reporting a 70% cost saving from an almost 50% reduction in individual testing [61] and a study in South Africa reporting a $1220 per 100 specimens (at $40 per test) saving from a 30% reduction in individual testing [62].

#### Reducing testing frequency

A recent costing study assessing the cost-effectiveness of viral load compared to CD4 testing determined that the cost-effectiveness of viral load testing was sensitive to the frequency of testing, with annual viral loads being more cost-effective than the currently recommended six-monthly viral load testing [63]. Currently, WHO guidelines recommend viral load testing every six months [16] but, in practice, testing frequency varies. In Malawi, viral load testing is recommended to be performed every two years, whereas in South Africa it is done annually. Another study, from South Africa, that assessed the optimal timing of viral load testing concluded that viral load testing done at three months post-ART initiation is associated with better outcomes than viral load testing performed at six months [24]. These results suggest that an initial viral load is beneficial for detecting early adherence problems. After this initial phase, once patients have adapted to taking ART and reached stable and durable viral suppression, less frequent viral load testing may be possible. Future research is needed in this regard.

#### Replacing immunological monitoring with virological monitoring

Clinical trials conducted so far have only assessed the added value of viral load monitoring, rather than evaluating the potential to use viral load testing to replace CD4 as a patient-monitoring strategy [5–7]. Future trials should consider comparing CD4 and viral load monitoring head-to-head, following patients for longer duration, so that the possibility of abandoning immunological monitoring may be considered. Further evidence is required to assess the benefit of CD4 monitoring above and beyond viral load, following baseline CD4 at initiation (including for patients who develop clinical problems and to guide decisions about cotrimoxazole or fluconazole prophylaxis).

#### Prioritizing patients for viral load

Even with the many potential opportunities for simplifying the provision and reducing the cost of viral load testing, roll out at national level may still need to be phased in. Scale up of a triggered viral load testing approach using a clear algorithm to identify patients with CD4 reductions of 30% or more, specific clinical signs and those with poor adherence may be one approach. The MSF programme in Zimbabwe, recognizing a severe under-detection of treatment failure in their setting, implemented such an algorithm and saw a substantial increase in the number of viral load tests requested and subsequent detection of cases eligible for second-line ART. Alternative approaches, where routine viral load testing may not yet be feasible, may include the introduction of viral load testing to assess early adherence, continuing subsequent monitoring with CD4; as a tool to detect virological failure prior to switching to a less toxic first-line regimen; to confirm treatment failure before switching to second-line ART; or to monitor ARV-treated pregnant women before birth and during breast-feeding to confirm viral suppression. Introducing viral load in such a phased manner may allow for the logistical and technical laboratory capacity to be firmly established before scaling up the service to support routine virological monitoring for all.

## Discussion

Routine virological monitoring for HIV-positive patients on ART is important for early detection of virological failure, preventing the development of drug-resistant mutations, identifying patients in need of intensive adherence support and accurately diagnosing treatment failure. Through these clinically advantageous outcomes, the use of first-line drugs may be preserved and transmission of viral strains that are both drug sensitive and drug resistant may be limited.

Use of viral load testing may facilitate task shifting and reduce the number of clinical visits required for the patient. A once yearly viral load test could be the only treatment efficacy monitoring test required, which, if undetectable, allows for a simplified follow-up of patients on ART through community-based models, in line with the current priorities of the WHO [64].

Despite clear benefits, virological monitoring, especially on a routine basis, is the exception in resource-limited settings. It is therefore important for both national and international guidelines to clearly recommend routine virological monitoring for all patients on ART as the standard of care and for donors and governments to fund the implementation of accurate and operationally proven testing platforms in a comprehensive manner. Importantly, operational research will be required to investigate which tests work better at different levels of the healthcare system and in different settings.

The implementation of point-of-care viral load testing will need to be accompanied by operational research to determine which system, or combination of systems, work best in which contexts. Further simplification of laboratory-based tests, so that they can be performed at district level, will allow for the decentralization of testing currently performed at national level and should thus be prioritized.

Ultimately, the price of viral load testing will have to be reduced to benefit the majority of patients in need. There are currently only four main suppliers of single-manufacturer viral load testing platforms, and just one of those has a majority stake in Africa [48]. These four platforms are expensive, require a high level of technical skill and laboratory infrastructure, and are more suited to national or reference laboratories. A broader availability of tests capable of being placed at district laboratory and clinic level, without the formation of a monopoly by a single manufacturer, is therefore required. Going forward, it will be important to ensure that multiple manufacturers are able to enter what will be a growing market for viral load testing and that incentives for manufacturers of quality-approved generics are encouraged through mechanisms, such as cooperative licensing strategies, that will enable access to the large number of overlapping patents applicable to molecular techniques [65]. Simplification of testing along with price-reducing strategies is needed to support full implementation of viral load monitoring in remote and resource-limited settings.
